# A machine learning approach to identify distinct subgroups of veterans at risk for hospitalization or death using administrative and electronic health record data

**DOI:** 10.1371/journal.pone.0247203

**Published:** 2021-02-19

**Authors:** Ravi B. Parikh, Kristin A. Linn, Jiali Yan, Matthew L. Maciejewski, Ann-Marie Rosland, Kevin G. Volpp, Peter W. Groeneveld, Amol S. Navathe

**Affiliations:** 1 Corporal Michael J. Crescenz Veterans Affairs Medical Center, Philadelphia, Pennsylvania, United States of America; 2 VA Center for Health Equity Research and Promotion, Pittsburgh, Pennsylvania, United States of America; 3 Department of Medical Ethics and Health Policy, Perelman School of Medicine, University of Pennsylvania, Philadelphia, Pennsylvania, United States of America; 4 Leonard Davis Institute of Health Economics, University of Pennsylvania, Philadelphia, Pennsylvania, United States of America; 5 Department of Biostatistics, Epidemiology and Informatics, Perelman School of Medicine, University of Pennsylvania, Philadelphia, Pennsylvania, United States of America; 6 Durham Center of Innovation to Accelerate Discovery and Practice Transformation, Durham Veterans Affairs Health Care System, Durham, North Carolina, United States of America; Juntendo University Urayasu Hospital, JAPAN

## Abstract

**Background:**

Identifying individuals at risk for future hospitalization or death has been a major priority of population health management strategies. High-risk individuals are a heterogeneous group, and existing studies describing heterogeneity in high-risk individuals have been limited by data focused on clinical comorbidities and not socioeconomic or behavioral factors. We used machine learning clustering methods and linked comorbidity-based, sociodemographic, and psychobehavioral data to identify subgroups of high-risk Veterans and study long-term outcomes, hypothesizing that factors other than comorbidities would characterize several subgroups.

**Methods and findings:**

In this cross-sectional study, we used data from the VA Corporate Data Warehouse, a national repository of VA administrative claims and electronic health data. To identify high-risk Veterans, we used the Care Assessment Needs (CAN) score, a routinely-used VA model that predicts a patient’s percentile risk of hospitalization or death at one year. Our study population consisted of 110,000 Veterans who were randomly sampled from 1,920,436 Veterans with a CAN score≥75^th^ percentile in 2014. We categorized patient-level data into 119 independent variables based on demographics, comorbidities, pharmacy, vital signs, laboratories, and prior utilization. We used a previously validated density-based clustering algorithm to identify 30 subgroups of high-risk Veterans ranging in size from 50 to 2,446 patients. Mean CAN score ranged from 72.4 to 90.3 among subgroups. Two-year mortality ranged from 0.9% to 45.6% and was highest in the home-based care and metastatic cancer subgroups. Mean inpatient days ranged from 1.4 to 30.5 and were highest in the post-surgery and blood loss anemia subgroups. Mean emergency room visits ranged from 1.0 to 4.3 and were highest in the chronic sedative use and polysubstance use with amphetamine predominance subgroups. Five subgroups were distinguished by psychobehavioral factors and four subgroups were distinguished by sociodemographic factors.

**Conclusions:**

High-risk Veterans are a heterogeneous population consisting of multiple distinct subgroups–many of which are not defined by clinical comorbidities–with distinct utilization and outcome patterns. To our knowledge, this represents the largest application of ML clustering methods to subgroup a high-risk population. Further study is needed to determine whether distinct subgroups may benefit from individualized interventions.

## Introduction

Approximately 5% of the US population account for a disproportionate share of hospitalizations and deaths [1, 2]. This phenomenon is also present among Veterans seeking care within the Veterans Health Administration (VA) [3]. Identifying patients at risk for impending hospitalization or death has been a key priority for the VA, owing to the disproportionate share of health care spending among a small percentage of Veterans who are hospitalized and/or die [3–5]. To enhance prediction, the VA developed the Care Assessment Needs (CAN) score, which accurately predicts risk of future hospitalization or death for over 5 million Veterans annually who receive VA primary care [6]. However, individuals at high risk for hospitalization or death are a heterogeneous group often characterized by a high prevalence of adverse social factors, such as inadequate transportation or housing [7, 8]. One-size-fits all interventions–including telemedicine or case management programs–have had limited effectiveness in improving outcomes among high-risk individuals, and no standardized programs exist for Veterans with high CAN scores [9–12]. Thus, there has been considerable effort to identify subgroups of high-risk individuals, in order to inform the development of targeted strategies with greater chances of effectiveness [7, 8, 13].

Prior approaches to subgrouping high-risk patients (patients identified by prediction models as high-risk for poor outcomes) have relied on limited diagnosis and/or disease criteria and/or expert opinion that is prone to human error [14]. Detailed encounter data in electronic health records (EHRs) may give broader insights into patterns of health care use and disease severity [15, 16]. Additionally, including data on adverse sociodemographic and psychobehavioral factors, which may lead to differing patterns in health care utilization and explain poorer outcomes among minority groups, may also improve the ability to identify subgroups of patients [17]. Advances in computational capacity and machine learning now allow for clustering of distinct subgroups using several or all of these data sources simultaneously [18]. Using granular clinical and socioeconomic data with machine learning approaches for subgrouping could facilitate the development of more efficacious intervention strategies that target the distinct needs of specific, homogeneous patient populations at high risk for hospitalization or death.

The purpose of this study was to examine whether machine learning algorithms could identify distinct subgroups of high-risk patients. We empirically derived subgroups from a large population of high-risk Veterans using a database that merges administrative and detailed EHR data to access encounter-related, laboratory and vital sign data, and socioeconomic data that are not commonly used to subgroup high-risk patients. We also assessed patterns of utilization and mortality within each of these clusters. We hypothesized that, while most subgroups would be differentiated by comorbidity-based factors, a small number of subgroups would be defined by non-comorbidity-based factors including psychobehavioral or sociodemographic factors. Such subgroups would have likely not been identified using only clinical data, missing an opportunity to better understand the needs of these subgroups and potentially to provide more personalized services.

## Methods

### Overview

In this retrospective cohort study, we derived distinct subgroups of high-risk Veterans using a machine learning (density-based clustering) technique, which has previously been shown to identify more distinct clinical subgroups than connectivity-based or centroid-based clustering algorithms [16]. Then, we used a set of discriminative models to determine which variables best characterized each cluster, allowing us to meaningfully label each cluster. The Corporal Michael J. Crescenz VA Medical Center Institutional Review Board approved this study with a waiver of informed consent.

### The Care Assessment Needs score

To define a high-risk population, we used the CAN score to select our study’s cohort because it is implemented and available nationwide in the VA. Although we use additional data (e.g., individual socioeconomic variables) beyond what is used in the CAN score model in clustering Veterans into subgroups, we defined high-risk Veterans using the CAN score to tightly align with current VA operations. First developed in 2010 to proactively identify Veterans at high risk for poor outcomes, the CAN score uses routinely collected EHR and administrative data from the VA Corporate Data Warehouse (CDW) to generate weekly estimates of hospitalization and/or mortality risk for the over 5 million Veterans who have at least one primary care visit through the VA annually [6, 19, 20]. CAN scores are obtained by transforming probabilities of hospitalization and/or death to percentiles of risk within the VA primary care population. For example, an individual with a one-year composite CAN score of 95 has a risk of hospitalization or death within one year that is greater than 95% of the VA primary care population or over a 25% predicted risk of hospitalization. The CAN score is accessed by approximately 1,000 VA clinicians, care managers, and other staff nationwide at least 5,000 times every month [19, 21]. We used a version of the CAN score (CAN 2.0) that was based on 36 variables and has good predictive performance (area under the curve 0.82).

### Population

To focus on a cohort of Veterans who received VA-based primary care (i.e., Veterans who are most relevant to VA health care prediction models) and who thus had an associated CAN score, we obtained data from the VA Primary Care Management Module (PCMM), which identifies patients assigned to VA primary care clinicians. To define the high-risk population, we used the one-year composite CAN score and obtained all of the weekly CAN scores from January 1, 2014, to December 31, 2014. Any patient who had a weekly CAN score ≥75 at any point in 2014 was considered high-risk (*n* = 1,920,436). We chose to use 2014 data so that we could measure two-year outcomes from 2015–2016. CAN ≥75 is a recognized marker of high risk of hospitalization or death and encompasses patients who may be considered in a second tier of risk, but who have potentially more modifiable risk factors [22]. We excluded patients who died during 2014 (*n* = 109,881) to simulate the cohort of high-CAN patients that would be able to be identified and clustered on December 31, 2014.

**Fig 1:**
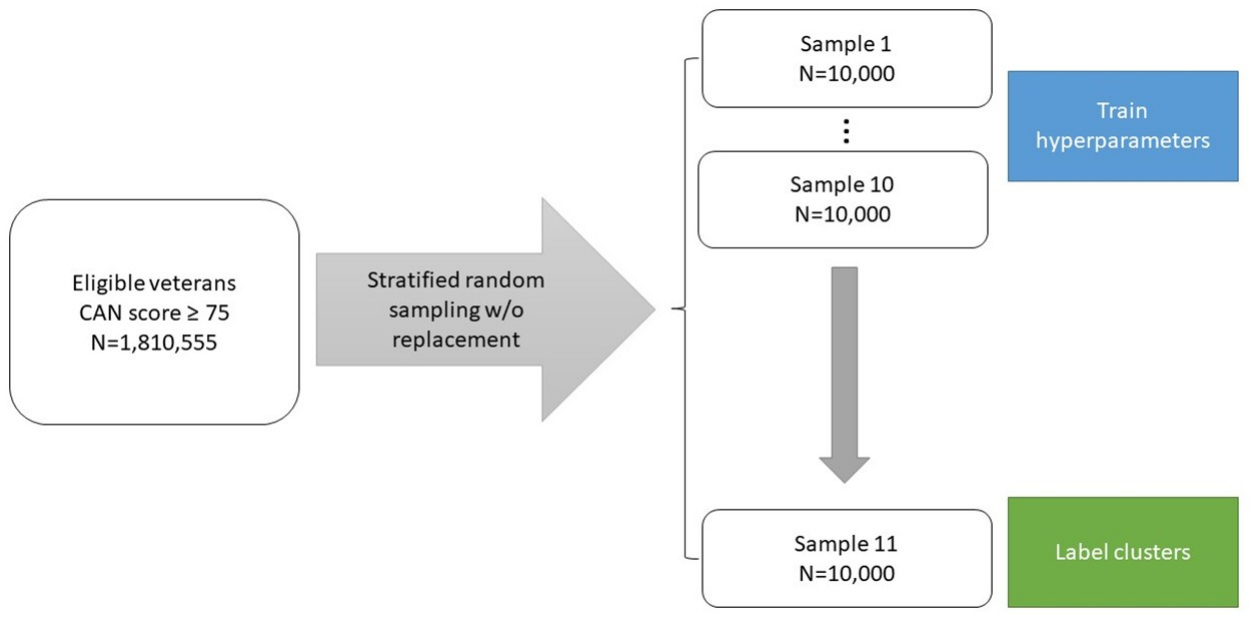
Study schematic.

From the cohort of 1,810,555 eligible patients, we randomly sampled 11 independent data sets without replacement from the study population, each of size n = 10,000. Subsampling was required because of the computational constraints of the VA Informatics and Computing Infrastructure (VINCI) network that housed the data. Each random sample was selected by stratifying the CAN score in 5-point increments so that the sample distribution reflected the population distribution of CAN scores (see S1 Fig). The study cohort thus consisted of 110,000 Veterans. Of the 11 data sets, 10 were used for training to determine model hyperparameters; and 1 was used to obtain the final set of clusters, which were identified and labelled (see Fig 1).

### Data source and features for clustering

We used data from the VA CDW, a national repository of administrative and electronic health data containing inpatient, outpatient, laboratory, procedure, and pharmacy encounters for all Veterans who seek any care within any VA medical center or outpatient clinic; all data elements have been validated and are routinely used in research [20, 23–27]. There are no routine exclusions of Veterans from the CDW, although non-VA care received is not routinely captured in the CDW. The CDW also captures important data related to type of encounter, in order to differentiate between specific types of outpatient visits (home-based, phone-based, face-to-face), Medicaid eligibility, and race.

We used 119 variables as features for clustering (see S1 Table), based upon six domains (sociodemographic and insurance features, comorbidities, pharmacy (including weighted medication adherence [28]), vital signs, laboratories, and prior utilization) that are routinely available through the CDW. Of note, many of our features are included in the CAN 2.0 score. However, we also included several features that may be indicative of adverse socioeconomic status, including race, ethnicity, insurance status, medication adherence (defined as the proportion of days covered (PDC) among a limited set of common outpatient medications), and detailed encounter data (e.g. number of psychiatry visits) that are not present in the CAN score. Only data from January 1 through December 31, 2014, were used for clustering. For data with multiple observations, we used the medians as features to reduce noise. In order to select relevant features, we removed 296 of 415 original variables with extremely low variance and those that were highly correlated (defined as r > 0.80). We arrived at a final variable set of 119 variables. To address missing variables, we 1) imputed all missing variables a single time using Fully Conditional Specification, and 2) created a separate variable indexing the count of missing variables out of the final variable set (see S1 File).

### Dimension reduction and clustering

We then utilized t-distributed stochastic neighbor embedding (t-SNE) to find a two-dimensional representation of all features. t-SNE is a non-linear dimension reduction technique that attempts to preserve the structure of pairwise similarities of the original observations in the lower-dimensional representation. We performed t-SNE separately on each of the 11 data sets [29, 30]. We opted to use t-SNE after exploring other dimension reduction techniques (see S1 File). Based on previous work, we then used the Ordering Points To Identify the Clustering Structure (OPTICS) algorithm–a density-based clustering algorithm—on each t-SNE dimension-reduced data set to achieve the best performance [31]. Additional details are provided in the S1 File.

### Identifying subgroups

While density-based clustering algorithms may identify clusters of similar individuals, providing interpretable descriptions of the clusters is challenging. Univariate summaries that compare the within- and between-cluster variation explained by each clustering feature fail to account for correlation among features. We used a multivariate, supervised learning approach to describe clusters. Specifically, we employed a set of ridge regression models, applied in a one-versus-rest manner (i.e., with the outcome being a binary indicator of membership in a given cluster [1] versus any other cluster [0]), to better understand the cluster assignments in the validation set. We fit 100 models for each cluster corresponded to 100 values of ridge penalty parameter, where the dependent variable was a dichotomous indicator of assignment to a given cluster (1) versus assignment to any of the remaining clusters (0), and the independent variables were the original 119 variables. After averaging the ridge penalty parameter for each cluster and normalization of the estimates, the magnitudes of the resulting model coefficient estimates represent the relative contribution of each independent variable to discriminating patients in one cluster from other high-risk patients. Following model fitting, we computed the range of the estimated coefficients (maximum value minus minimum value) across clusters for each independent variable. This metric is a measure of variable importance, where a large value suggests that the variable is useful for distinguishing observations in one cluster from the rest. In order to visualize these results, we plotted the variables with the largest 20 ranges for each of the clustering methods. Finally, four authors (RBP, JY, KAL, ASN)–two of whom are VA clinicians–used the ridge regression coefficients to assign a label to each of the clusters. Details are provided in the S1 File.

### Outcomes

We descriptively examined several two-year (2015–2016) outcomes for each cluster identified via machine learning methods: rate of any-cause hospitalization within VA hospitals, the number of days hospitalized at any VA hospital, rate of any-cause emergency department (ED) visits within VA hospitals, the number of emergency department visits within VA hospitals, and all-cause mortality rate using the VA Vital Status file, which uses the Social Security Administration Death Master File plus internal VA records to assess mortality [32]. While all hospitalization and ED data only assesses utilization within VA hospitals, our mortality outcome captured deaths that occurred in non-VA settings within the United States as well because we used national data on death.

## Results

### Baseline characteristics of high-risk Veterans

In a national population of high-risk Veterans (n = 1,810,555), the mean age was 64.7 years (standard deviation [SD] 14.1), 372,138 (20.6%) were Black, 10,258 (0.6%) were Hispanic, 128,693 (7.1%) were female, and 25,567 (1.5%) were on Medicaid; these statistics were similar for the 110,000 Veteran sample used for clustering (see Table 1). High-risk Veterans had an average Elixhauser comorbidity score of 3.3 (SD 7.9), the most common of which were depression (40.8%), diabetes without complications (36.9%), and chronic pulmonary disease (26.7%). 10.6% of the veterans also had drug abuse. High-risk Veterans had high rates of emergency room (42.0%) and inpatient (19.1%; 13.2% general medicine; 6.2% surgical) utilization. 92.7% had documented use of at least one medication (62.0% antihypertensives, 40.7% antidepressants, 28.4% glucose-lowering agents).

**Table 1:** Baseline population characteristics.

	CAN score ≥75	Subsample
n	1,810,555	110,000
Age (mean, SD)	64.68 (14.12)	64.74 (14.13)
Female (n, %)	128693 (7.1)	7840 (7.1)
Race (n, %)		
Non-Hispanic White	1289373 (71.2)	78354 (71.2)
Black	372138 (20.6)	22566 (20.5)
Hispanic/Latino	10258 (0.6)	601 (0.5)
Other	138786 (7.7)	8479 (7.7)
Medicaid (n, %)	25567 (1.5)	1520 (1.4)
Comorbidities		
Elixhauser groups (mean, SD)	3.71 (2.26)	3.71 (2.26)
Elixhauser score (mean, SD)	3.26 (7.87)	3.25 (7.88)
CHF (n, %)	188401 (10.4)	11321 (10.3)
Chronical Pulmonary Disease (n, %)	484106 (26.7)	29344 (26.7)
Diabetes without complications (n, %)	667226 (36.9)	40626 (36.9)
Diabetes with complications (n, %)	242043 (13.4)	14935 (13.6)
Metastatic cancer (n, %)	26059 (1.4)	1614 (1.5)
Drug abuse (n, %)	191039 (10.6)	11519 (10.5)
Depression (n, %)	737906 (40.8)	44954 (40.9)
Outpatient utilization in 2014 (n, %)		
Any outpatient visit	1798833 (99.4)	109308 (99.4)
Emergency room	759563 (42.0)	45954 (41.8)
Primary care	1754218 (96.9)	106649 (97.0)
Psychiatry	749752 (41.4)	45423 (41.3)
Specialty care	1623823 (89.7)	98515 (89.6)
Inpatient utilization in 2014 (n, %)		
Any inpatient visit	346559 (19.1)	21129 (19.2)
General medicine	239750 (13.2)	14687 (13.4)
Surgery	111547 (6.2)	6836 (6.2)
Laboratories (mean, SD)		
Albumin	3.93 (0.41)	3.93 (0.41)
Creatinine	1.11 (0.42)	1.11 (0.41)
Hemoglobin A1C	6.62 (1.41)	6.63 (1.42)
Vital signs (mean, SD)		
Systolic blood pressure	132.77 (15.12)	132.82 (15.16)
Diastolic blood pressure	76.39 (9.50)	76.34 (9.51)
BMI	29.87 (6.37)	29.88 (6.36)
Medication use (n, %)		
Any medication	1677705 (92.7)	101854 (92.6)
Antidepressants	737559 (40.7)	44941 (40.9)
Antihypertensives	1122708 (62.0)	68174 (62.0)
Glucose lowering agents	514748 (28.4)	31289 (28.4)
Mean weighted medication adherence (mean, SD)	0.77 (0.22)	0.77 (0.22)

### Subgroups

**Fig 2:**
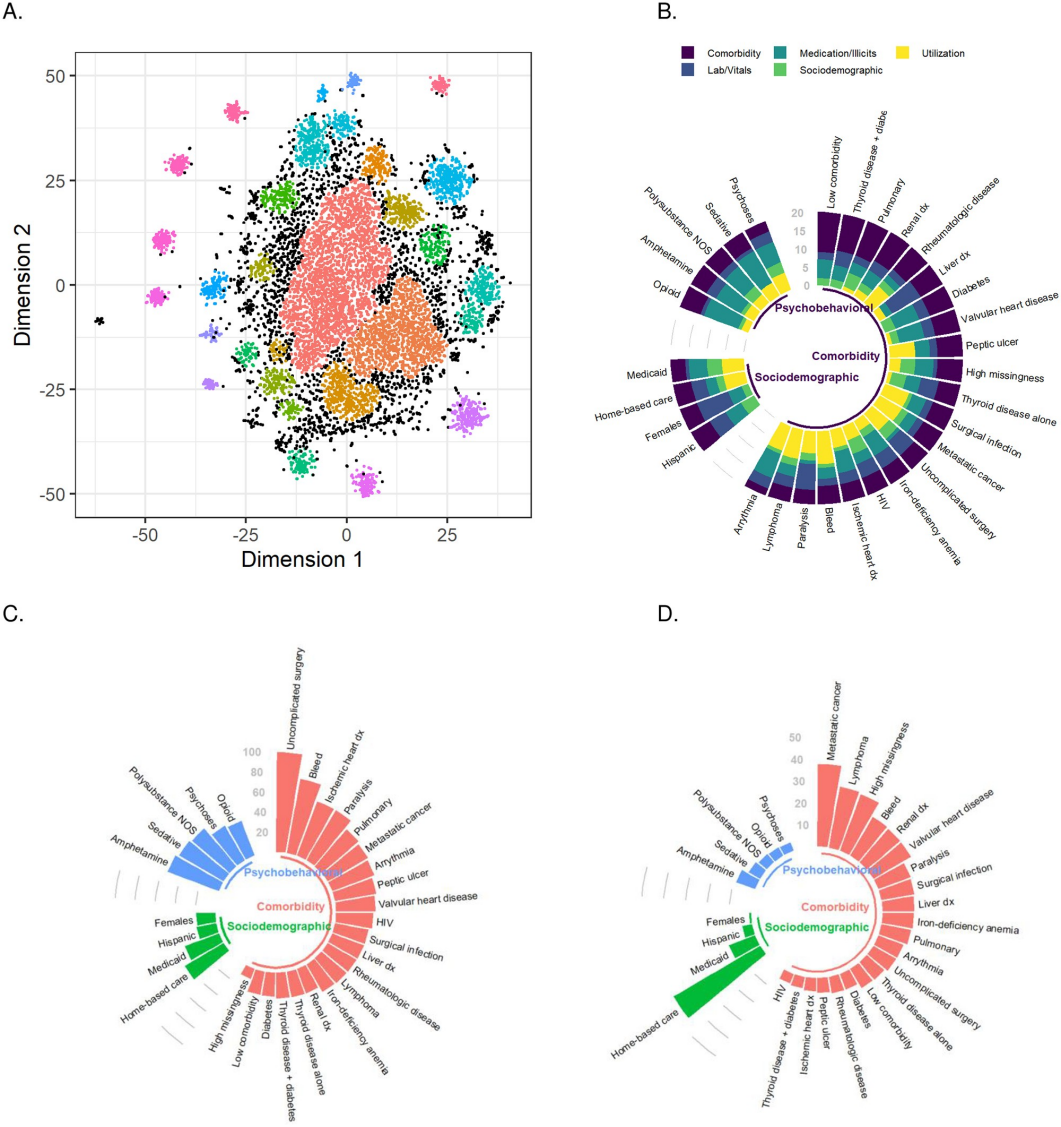
(A) t-SNE plot in validation set. (B) Distribution of variables contributing to each cluster. (C) Two-year inpatient hospitalization rate across clusters. (D) Two-year mortality across clusters.

Cluster analysis revealed 30 distinct subgroups of high-risk Veterans (see Fig 2A). 74.8% of high-risk Veterans were clustered into one of these subgroups, and 25.2% of high-risk Veterans were not clustered. These clusters are broadly described in Table 2. Size of clusters varied from 50 (0.5%, *polysubstance use–amphetamine predominant*) to 2,446 (24.5%, *low comorbidity*) (see S2 Table). Twenty-one out of thirty subgroups were distinguished by non-psychiatric chronic conditions (e.g. *metastatic cancer*). Five subgroups (*psychoses without substance use* and four subgroups of polysubstance use [*opioid predominant*, *amphetamine predominant*, *sedative predominant*, and *not otherwise specified*) were distinguished by psychobehavioral factors. Four groups were distinguished by sociodemographic factors, including utilization-related (e.g. *home-based care*) and demographic (e.g. *Medicaid predominant*, *female predominant*) factors. One cluster was identified by a high prevalence of missing data (*high missingness*) (see Fig 2B).

**Table 2:** Clusters identified in validation set.

Category	Label	n
Comorbidity	Low comorbidity burden	2446
Comorbidity	Insulin-dependent diabetes	1156
Comorbidity	Chronic renal disease	390
Comorbidity	Ischemic heart disease	50
Comorbidity	High Missingness	249
Comorbidity	Uncomplicated surgery	84
Comorbidity	Valvular heart disease	136
Comorbidity	Pulmonary vascular disease	65
Comorbidity	Chronic liver disease	157
Comorbidity	Iron-deficiency anemia	70
Comorbidity	Cardiac arrhythmias	115
Comorbidity	Thyroid disease with diabetes	115
Comorbidity	Thyroid disease without diabetes	190
Comorbidity	Metastatic cancer	128
Comorbidity	Paralysis/spinal cord injuries	64
Comorbidity	Blood-loss anemia	54
Comorbidity	Rheumatologic disease	213
Comorbidity	Lymphoma without inpatient utilization	103
Comorbidity	Peptic ulcer disease	119
Comorbidity	HIV/AIDS	108
Comorbidity	Post-surgical infection	91
Psychobehavioral	Polysubstance use—amphetamine predominant	65
Psychobehavioral	Psychoses without polysubstance use	150
Psychobehavioral	Polysubstance use—not otherwise specified	273
Psychobehavioral	Polysubstance use—opioid predominant	116
Psychobehavioral	Polysubstance use—sedative predominant	50
Sociodemographic	Home-based care	136
Sociodemographic	Medicaid predominant	117
Sociodemographic	Hispanic predominant	154
Sociodemographic	Female predominant	320

The top five variables that distinguished each cluster are described in S3 Table. Generally, vital signs and laboratory data were less important in distinguishing subgroups than other variables. Age (47.2 to 77.5), composition of non-Hispanic White race (0.0% to 94.0%), mean number of comorbidities (0.5 to 6.7), and emergency room (7.6% to 79.6%) and inpatient (0.0% to 96.5%) utilization varied considerably across all clusters (see S4 Table).

### Utilization

The percentage of patients with an inpatient VA hospitalization in the two-year follow-up period ranged from 10.5% (the *high missingness* subgroup) to 98.6% (*uncomplicated surgery*) across all clusters (see Fig 2C). The clusters with the highest levels of inpatient hospitalization were *uncomplicated surgery* (98.6%), *blood-loss anemia* (74.4%), and *ischemic heart disease* (57.4%) (see S4 Table). The clusters with the lowest levels of inpatient hospitalization were *female predominant* (18.9%) and *Hispanic predominant* (19.7%).

The percentage of patients with an emergency room admission in the two-year follow-up period ranged from 28.2% (*high missingness*) to 76.7% (*blood-loss anemia*) across all clusters (see S4 Table). The clusters with the highest levels of emergency room admission were *blood-loss anemia* (76.7%), *polysubstance use–sedative predominant* (70.2%), and *pulmonary vascular disease* (64.3%). The clusters with the lowest levels of emergency room admission were *home-based care* (35.1%) and *paralysis/spinal cord injuries* (35.1%).

### Mortality

The percentage of patients who died during the two-year follow-up period ranged from 0.9% (*Female predominant*) to 45.6% (*home-based care*) across all clusters (see Fig 2D). The clusters with the highest mortality were *home-based care* (45.6%), *metastatic cancer* (37.5%), and *lymphoma without inpatient utilization* (28.2%) (see S4 Table). The clusters with the lowest mortality were *Female predominant* (0.9%), *psychoses without substance use* (4.0%), and *polysubstance use–opioid predominant* (4.3%).

## Discussion

In this retrospective cohort study of patients identified as high-risk by a predictive algorithm, we found substantial heterogeneity in subgroups of high-risk patients using a density-based clustering algorithm and variability in health care utilization (inpatient admission range 10.5–98.6%) and mortality (range 0.9–45.6%). Four of 30 subgroups were identified by predominantly sociodemographic rather than clinical features. To our knowledge, this is one of the first applications of machine learning to subgroup high-risk patients using EHR-enriched administrative data.

We identified 30 distinct subgroups among a heterogeneous national population of high-risk Veterans who receive care within the VA. Several clusters were identified by non-comorbidity-based -factors, including sociodemographic (e.g. high Medicaid and/or Hispanic predominant) and psychobehavioral (e.g. polysubstance abuse, psychoses) characteristics. Compared to existing work, this study has several unique strengths. Many traditional methods of defining high-risk individuals rely on pre-specifying the number of subgroups or defining risk using expert-driven frameworks [14], which may not account for certain factors, including health care use patterns or adverse social factors, that contribute to risk. In contrast, our clustering methods did not pre-specify numbers of subgroups and indeed did not cluster nearly 25.2% of all Veterans-. This ensured greater homogeneity within the identified subgroups. The identification of subgroups identified by comorbidity-based or non-comorbidity-based features provides a greater opportunity to develop, test, and implement tailored interventions. Identifying homogenous subgroups of high-risk populations, whether related to comorbidity-based, demographic, or psychobehavioral characteristics, is a critical first step.

Our clustering utilized both routinely collected administrative data and detailed utilization and laboratory data. Some identified subgroups have been previously described in other work to cluster high-risk individuals, such as liver disease, congestive heart failure, and renal disease clusters [15]. By utilizing richer data related to utilization, laboratory values, and pharmacy data that may vary over time, we were able to identify distinct subgroups that have not routinely been identified by traditional methods and were characterized by sociodemographic characteristics. As an example, we identified a group characterized by a high prevalence of home-based care that would not have been separately identified if utilization data was not used to define subgroups. This group had high mortality but relatively low inpatient or emergency room utilization. Management strategies would likely differ between the home-based care subgroup–who may have low mobility and cognitive deficits and for whom efforts such as hospice and telemedicine may be prioritized–and a subgroup with high utilization but low mortality, such as the uncomplicated surgery subgroup–where counselling prior to readmission may be prioritized. Thus, our insights may have operational relevance within the healthcare system by using a routinely-used, prospective method to identify high-risk individuals.

As another example, we identified several clusters characterized by psychobehavioral factors. We identified four clusters of polysubstance abuse—sedative predominance, amphetamine predominance, opioid predominance, and not otherwise specified–with distinct patterns of inpatient utilization and mortality, since patients using sedatives and amphetamines had higher rates of inpatients utilization (54.2%, 51.1%) than the opioid group (36.9%) and higher mortality (9.2%, 6.0% vs. 4.3%). Current case management approaches to polysubstance abuse have been primarily based on those with opioid use disorder [33]. Our results suggest that groups characterized by predominantly sedative or amphetamine use have distinct patterns of outcomes and may warrant targeted substance use disorder programs.

Another significant advantage of our clustering method is that it does not assign every high-risk Veteran to a subgroup. Several clustering algorithms, including k-means and hierarchical clustering, require assignment to a cluster; thus, there may be considerable heterogeneity within a cluster due to a high predominance of outliers. Of note, other clustering methods, including latent class analysis, also do not. Indeed, 25.2% of our cohort was identified as an outlier–an individual who, potentially due to unique combinations of comorbidities, utilization patterns, and/or other features–was not sufficiently similar to other members of a cluster. These represent individuals who may have characteristics that are shared across multiple subgroups or are not captured (e.g. other sociobehavioral characteristics), potentially necessitating increased diversity in treatment programs to meet varying treatment needs. Identifying these outliers is important, as care management solutions can be costly and resource-intensive to deploy on a population-wide level. This may illustrate the need for additional survey data to unmask Veterans who are not easily subgrouped. While our approach may identify outliers who need to be further characterized, there may be logistical limitations given the relatively large proportion of individuals who were unable to be clustered.

These findings have important implications for the design of programs targeted at high-risk or high-needs populations. Currently, the VA and other organizations often suggest a range of varied interventions for high-risk individuals. Unsupervised learning could inform which subgroups of high-risk patients have distinct characteristics or patterns of utilization. While all will not be targetable, certain care interventions may be more appropriate for certain subgroups over others. Furthermore, these subgroups could facilitate testing and randomized experimentation of programs for specific subgroups, so-called “precision delivery”, in order to test hypotheses about care delivery [34, 35]. Having prior knowledge of Veteran population sub-structures could benefit the estimation of optimal intervention strategies using statistical methods for precision medicine.

There are several limitations to this analysis. First, this analysis was performed among a cohort of Veterans and may be difficult to generalize to non-Veterans. However, our population represented a diverse cohort with a variety of comorbidities and is representative of high-risk individuals across the US [6, 22]. Second, we did not have access to non-VA data, limiting our ability to examine utilization outside of the VA. However, our data source is representative of what is used in standard VA operations to identify high-risk individuals. Third, computational constraints required us to limit our sample to 110,000 Veterans. However, we took several steps to ensure generalizability across the entire Veteran population, including employing a stratified sampling strategy to reflect the CAN distribution across the VA and using a number of representative training sets to tune model parameters before clustering a final representative subsample of Veterans. Fourth, we did not have access to several relevant socioeconomic covariates (e.g., income, education level), although we did include indirect metrics such as enrollment priority and Medicaid status. We specifically did not include zip- or area-level socioeconomic metrics because there is significant heterogeneity in socioeconomic outcomes in these areas, and thus we limited ourselves to available individual-level data. Fifth, several identified clusters were relatively small, representing <1% of the population. It is unclear whether such subgroups would be large enough to warrant an individual system-wide program. Finally, while we used more detailed socioeconomic data to subgroup high-risk Veterans, our identification of high-risk Veterans was based on primarily clinical data. However, we used a standard operational algorithm to identify such high-risk Veterans in order to ensure operational relevance.

## Conclusion

Machine learning clustering identified several distinct subgroups of high-risk Veterans that were characterized by different clinical and/or socioeconomic characteristics along with differential utilization and mortality. This is one of the first applications of advanced clustering algorithms to subgroup a high-risk population using both administrative and EHR data. Future analyses should validate these subgroups in other Veteran and non-Veteran populations. As “one-size-fits-all” care management programs have been shown to have limited success among high-risk individuals, there is a need for targeted strategies for specific groups of high-risk individuals identified from a broad set of demographic, clinical and utilization features. The types of clustering methods applied in this analysis may serve as an important foundational step.

## Supporting information

S1 Table Variable list. (DOCX)

S2 Table Characteristics and outcomes of clusters. (DOCX)

S3 Table Comparison of 5 most important variables that contribute to clusters within validation cohort. (DOCX)

S4 Table Outcomes (2015–2016) by cluster. (DOCX)

S1 Fig Distribution of CAN scores in cohort. (DOCX)

S1 File (DOCX)
