# Supplementary material for: A machine learning approach to identify distinct subgroups of veterans at risk for hospitalization or death using administrative and electronic health record data
Source: PLoS One. 2021 Feb 19;16(2):e0247203. doi: 10.1371/journal.pone.0247203 (PMC7894856; doi:10.1371/journal.pone.0247203)
Supplement: S1 Table — (DOCX) [file pone.0247203.s001.docx]

**S1 Table. Variable List**

| **Variable name** | **Label** | **Category** | **Used in clustering algorithm** | **% missing in high risk group** | **% missing in validation set** | **Social Factor** |
| --- | --- | --- | --- | --- | --- | --- |
| gender | gender | demographics | Y | 0% | 0% | Y |
| age2014 | Age as of 1/1/2014 | demographics | Y | 0% | 0% | Y |
| ethnicity2 | ethnicity | demographics | Y | 0% | 0% | Y |
| race3 | race group | demographics | Y | 0% | 0% | Y |
| cEvent_1y14 | CANscore 2014 | demographics | Y | 0% | 0% |  |
| EnrollPriority | Enrollment Priority | demographics | Y | 0% | 0% | Y |
| ServiceConnectedPercentage | Service Connected Percentage | demographics | Y | 0% | 0% | Y |
| ExposedToAgentOrangeFlag | Agent Orange Exposure | demographics | Y | 0% | 0% | Y |
| MedicaidFlag | Medicaid Beneficiary | demographics | Y | 3% | 3% | Y |
| 1_ELIX_CONGESTIVE_HEART_FAILURE | Congestive Heart Failure | comorbidities | Y | 0% | 0% |  |
| 2_ELIX_CARDIAC_ARRHYTHMIA | Cardiac Arrhythmia | comorbidities | Y | 0% | 0% |  |
| 3_ELIX_VALVULAR_DISEASE | Valvular Disease | comorbidities | Y | 0% | 0% |  |
| 4_ELIX_PULMONARY_CIRCULATION_DISEASE | Pulmonary Circulation Disease | comorbidities | Y | 0% | 0% |  |
| 5_ELIX_PERIPHERAL_VASCULAR_DISORDER | Peripheral Vascular Disorder | comorbidities | Y | 0% | 0% |  |
| 6_ELIX_HYPERTENSION_UNCOMPLICATED | Uncomplicated Hypertension | comorbidities | Y | 0% | 0% |  |
| 7_ELIX_HYPERTENSION_COMPLICATED | Complicated Hypertension | comorbidities | Y | 0% | 0% |  |
| 8_ELIX_PARALYSIS | Paralysis | comorbidities | Y | 0% | 0% |  |
| 9_ELIX_OTHER_NEUROLOGICAL_DISORDERS | Other Neurological Disorders | comorbidities | Y | 0% | 0% |  |
| 10_ELIX_CHRONIC_PULMONARY_DISEASE | Chronic Pulmonary Disease | comorbidities | Y | 0% | 0% |  |
| 11_ELIX_DIABETES_UNCOMPLICATED | Uncomplicated Diabetes | comorbidities | Y | 0% | 0% |  |
| 12_ELIX_DIABETES_COMPLICATED | Complicated Diabetes | comorbidities | Y | 0% | 0% |  |
| 13_ELIX_HYPOTHYROIDISM | Hypothyroidism | comorbidities | Y | 0% | 0% |  |
| 14_ELIX_RENAL_FAILURE | Renal Disease | comorbidities | Y | 0% | 0% |  |
| 15_ELIX_LIVER_DISEASE | Liver Disease | comorbidities | Y | 0% | 0% |  |
| 16_ELIX_PEPTIC_ULCER_DISEASE_EXCLUDING_BLEEDING | Peptic Ulcer Disease | comorbidities | Y | 0% | 0% |  |
| 17_ELIX_AIDS_HIV | HIV/AIDS | comorbidities | Y | 0% | 0% |  |
| 18_ELIX_LYMPHOMA | Lymphoma | comorbidities | Y | 0% | 0% |  |
| 19_ELIX_METASTATIC_CANCER | Metastatic Solid Tumors | comorbidities | Y | 0% | 0% |  |
| 20_ELIX_SOLID_TUMOR_WITHOUT_METASTASIS | Non-metastatic Solid Tumors | comorbidities | Y | 0% | 0% |  |
| 21_ELIX_RHEUMATOID_ARTHRITIS_COLLAGEN | Rheumatologic Disease | comorbidities | Y | 0% | 0% |  |
| 22_ELIX_COAGULOPATHY | Coagulopathy | comorbidities | Y | 0% | 0% |  |
| 23_ELIX_OBESITY | Obesity | comorbidities | Y | 0% | 0% |  |
| 24_ELIX_WEIGHT_LOSS | Weight Loss | comorbidities | Y | 0% | 0% |  |
| 25_ELIX_FLUID_AND_ELECTROLYTE_DISORDERS | Fluid and Electrolyte Disorders | comorbidities | Y | 0% | 0% |  |
| 26_ELIX_BLOOD_LOSS_ANEMIA | Blood Loss Anemia | comorbidities | Y | 0% | 0% |  |
| 27_ELIX_DEFICIENCY_ANEMIA | Deficiency Anemia | comorbidities | Y | 0% | 0% |  |
| 28_ELIX_ALCOHOL_ABUSE | Alcohol Abuse | comorbidities | Y | 0% | 0% | Y |
| 29_ELIX_DRUG_ABUSE | Drug Abuse | comorbidities | Y | 0% | 0% | Y |
| 30_ELIX_PSYCHOSES | Psychoses | comorbidities | Y | 0% | 0% |  |
| 31_ELIX_DEPRESSION | Depression | comorbidities | Y | 0% | 0% |  |
| 1_OTHER_GASTROPARESIS | Gastroparesis | comorbidities | Y | 0% | 0% |  |
| 2_OTHER_ANXIETY | Anxiety | comorbidities | Y | 0% | 0% |  |
| 3_OTHER_SEDATIVES | Sedative Use | comorbidities | Y | 0% | 0% | Y |
| 4_OTHER_AMPHETAMINES | Amphetamine Use | comorbidities | Y | 0% | 0% | Y |
| 5_OTHER_HALLUCINOGENS | Hallucinogen Use | comorbidities | Y | 0% | 0% | Y |
| 6_OTHER_OPIODS | Opiod Use | comorbidities | Y | 0% | 0% | Y |
| 7_OTHER_COCAINE | Cocaine Use | comorbidities | Y | 0% | 0% | Y |
| 8_OTHER_CANNABIS | Cannabis Use | comorbidities | Y | 0% | 0% | Y |
| 9_OTHER_TOBACCO | Tobacco Use | comorbidities | Y | 0% | 0% | Y |
| inpt_surgery_sum | Inpatient surgery visits | utilization | Y | 0% | 0% |  |
| inpt_cardiology_sum | Inpatient cardiology visits | utilization | Y | 0% | 0% |  |
| inpt_medical_sum | Inpatient medical visits | utilization | Y | 0% | 0% |  |
| inpt_nursing_rehab_sum | Inpatient nursing rehab visits | utilization | Y | 0% | 0% |  |
| inpt_psychiatric_sum | Inpatient psychiatric visits | utilization | Y | 0% | 0% |  |
| inpt_hospice_sum | Inpatient hospice visits | utilization | Y | 0% | 0% |  |
| inpt_spinal_cord_sum | Inpatient spinal cord visits | utilization | Y | 0% | 0% |  |
| inpt_neurology_non_sc_sum | Inpatient non-spinal cord neurology visits | utilization | Y | 0% | 0% |  |
| inpt_oncology_sum | Inpatient oncology visits | utilization | Y | 0% | 0% |  |
| inpt_substanceuse_sum | Inpatient substance use visits | utilization | Y | 0% | 0% | Y |
| out_card_sum | Outpatient cardiology visits | utilization | Y | 0% | 0% |  |
| out_dental_sum | Outpatient dental visits | utilization | Y | 0% | 0% |  |
| out_er_sum | Outpatient ER visits | utilization | Y | 0% | 0% |  |
| out_neuro_sum | Outpatient neurology visits | utilization | Y | 0% | 0% |  |
| out_nonface_sum | Outpatient non-face-to-face visits | utilization | Y | 0% | 0% | Y |
| out_onc_sum | Outpatient oncology visits | utilization | Y | 0% | 0% |  |
| out_pc_sum | Outpatient primary care visits | utilization | Y | 0% | 0% |  |
| out_pcphonecare_sum | Outpatient primary care phone visits | utilization | Y | 0% | 0% |  |
| out_phonecare_sum | Outpatient phone visits | utilization | Y | 0% | 0% |  |
| out_psychiatric_sum | Outpatient psychiatric visits | utilization | Y | 0% | 0% |  |
| out_rad_sum | Outpatient radiology visits | utilization | Y | 0% | 0% |  |
| out_renal_sum | Outpatient nephrology visits | utilization | Y | 0% | 0% |  |
| out_substanceuse_sum | Outpatient substance use visits | utilization | Y | 0% | 0% |  |
| out_surg_sum | Outpatient surgery visits | utilization | Y | 0% | 0% |  |
| out_CT_sum | Outpatient CT visits | utilization | Y | 0% | 0% |  |
| out_MRI_sum | Outpatient MRI visits | utilization | Y | 0% | 0% |  |
| out_echo_sum | Outpatient echocardiography visits | utilization | Y | 0% | 0% |  |
| out_nuclear_sum | Outpatient nuclear radiology visits | utilization | Y | 0% | 0% |  |
| out_ultrasound_sum | Outpatient ultrasound visits | utilization | Y | 0% | 0% |  |
| out_xray_sum | Outpatient X-ray visits | utilization | Y | 0% | 0% |  |
| ALT_median | ALT median | laboratories | Y | 17% | 16% |  |
| ALBUMIN_median | Albumin median | laboratories | Y | 20% | 20% |  |
| ALKALINE_PHOSPHATASE_median | Alkaline Phosphatase median | laboratories | Y | 18% | 18% |  |
| AST_median | AST median | laboratories | Y | 16% | 15% |  |
| BILIRUBIN_median | Total Bilirubin median | laboratories | Y | 19% | 18% |  |
| CHOLESTEROL_LDL_median | LDL cholesterol median | laboratories | Y | 19% | 19% |  |
| CREATININE_median | Creatinine median | laboratories | Y | 9% | 9% |  |
| GLUCOSE_median | Glucose median | laboratories | Y | 10% | 9% |  |
| HEMOGLOBIN_median | Hemoglobin median | laboratories | Y | 14% | 14% |  |
| A1C_TOTAL_median | Hemoglobin A1C median | laboratories | Y | 35% | 34% |  |
| LEUKOCYTES_median | Leukocytes median | laboratories | Y | 16% | 16% |  |
| PLATELET_median | Platelet median | laboratories | Y | 16% | 16% |  |
| SODIUM_median | Sodium median | laboratories | Y | 8% | 8% |  |
| UREA_NITROGEN_median | Urea Nitrogen median | laboratories | Y | 11% | 11% |  |
| ANTIDEPRESSANTS_n | # of antidepressants | pharmacy | Y | 0% | 0% |  |
| ANTIINFLAM_RHEUMATIC_n | # of anti-inflammatory | pharmacy | Y | 0% | 0% |  |
| BETA_BLOCKERS_n | # of beta blockers | pharmacy | Y | 0% | 0% |  |
| LIP_MOD_AGENTS_PLAIN_n | # of lipid modifying agents | pharmacy | Y | 0% | 0% |  |
| OPIOIDS_n | # of opioids | pharmacy | Y | 0% | 0% | Y |
| OPIOIDS_THERAPY_n | # of opioid abuse therapies | pharmacy | Y | 0% | 0% | Y |
| ACE_INHIBITORS_PLAIN_n | # of ACE inhibitors | pharmacy | Y | 0% | 0% |  |
| DIURETICS_THIAZIDES_n | # of thiazide diuretics | pharmacy | Y | 0% | 0% |  |
| GLUCOSE_EXCL_INSULINS_n | # of non-insulin glucose lowering agents | pharmacy | Y | 0% | 0% |  |
| ANTIPSYCHOTICS_n | # of antipsychotics | pharmacy | Y | 0% | 0% |  |
| ANTITHROMBOTIC_n | # of antithrombotics | pharmacy | Y | 0% | 0% |  |
| ANTIINFECTIVES_n | # of antiinfectives | pharmacy | Y | 0% | 0% |  |
| INSULINS_AND_ANALOGUES_n | # of insulins | pharmacy | Y | 0% | 0% |  |
| THYROID_PREPARATIONS_n | # of thyroid agents | pharmacy | Y | 0% | 0% |  |
| ANTIARRHYTHMICS_n | # of antiarrhythmics | pharmacy | Y | 0% | 0% |  |
| weighted_pdc | Weighted medication adherence | pharmacy | Y | 0% | 0% |  |
| HEIGHT_median | Height median | vital signs | Y | 24% | 24% |  |
| WEIGHT_median | Weight median | vital signs | Y | 5% | 5% |  |
| PULSE_median | Heart rate median | vital signs | Y | 3% | 3% |  |
| PULSE_OXIMETRY_median | Pulse oximetry median | vital signs | Y | 13% | 12% |  |
| RESPIRATION_median | Respiratory rate median | vital signs | Y | 4% | 4% |  |
| DIASTOLIC_median | Diastolic BP median | vital signs | Y | 3% | 3% |  |
| SYSTOLIC_median | Systolic BP median | vital signs | Y | 3% | 3% |  |
| PAIN_median | Pain score median | vital signs | Y | 3% | 3% |  |
| TEMPERATURE_median | Temperature median | vital signs | Y | 4% | 4% |  |
| nMiss | Number of missing variables | demographics | Y | 0% | 0% |  |
| patienticn | patient identifier | demographics |  |  |  |  |
| veteranflag | veteran flag | demographics |  |  |  |  |
| dateofbirth | date of birth | demographics |  |  |  |  |
| age2014f | Age group | demographics |  |  |  |  |
| dateofdeath | date of death | demographics |  |  |  |  |
| dod16 | Date of death as end of FY16 | demographics |  |  |  |  |
| race | race | demographics |  |  |  |  |
| canscore75 | CANscore > 75? | demographics |  |  |  |  |
| canscore75dt | canscore75 date | demographics |  |  |  |  |
| canscore75_fy14 | CANscore > 75 in 2014? | demographics |  |  |  |  |
| riskDate14 | CANscore 2014 Date | demographics |  |  |  |  |
| canscore75_fy15 | CANscore > 75 in 2015? | demographics |  |  |  |  |
| cEvent_1y15 | CANscore 2015 | demographics |  |  |  |  |
| riskDate15 | CANscore 2015 Date | demographics |  |  |  |  |
| canscore75_fy16 | CANscore > 75 in 2016? | demographics |  |  |  |  |
| cEvent_1y16 | CANscore 2016 | demographics |  |  |  |  |
| riskDate16 | CANscore 2016 Date | demographics |  |  |  |  |
| ServiceConnectedFlag | Service Connected | demographics |  |  |  | Y |
| AidAndAttendanceFlag | Aid And Attendance Flag | demographics |  |  |  | Y |
| AnnualVACheckAmount | Annual VA Check Amount | demographics |  |  |  | Y |
| CatastrophicallyDisabledFlag | Catastrophically Disabled | demographics |  |  |  | Y |
| DisabilityRetirementFromMilitary | Disability Retirement From Military | demographics |  |  |  | Y |
| HouseboundBenefitsFlag | Housebound Benefits Flag | demographics |  |  |  | Y |
| MASEligibilitySID | MAS Eligibility | demographics |  |  |  | Y |
| MeansTestStatusSID | Means Test Status | demographics |  |  |  | Y |
| PowStatusIndicatedFlag | Prisoner of War | demographics |  |  |  | Y |
| PurpleHeartIndicatedFlag | Purple Heart | demographics |  |  |  | Y |
| RadiationExposureIndicatedFlag | Radiation Exposure | demographics |  |  |  | Y |
| RadiationExposureMethod | Radiation Exposure Method | demographics |  |  |  | Y |
| SouthwestAsiaConditionsFlag | Southwest Asia Conditions | demographics |  |  |  | Y |
| UnemployableFlag | Unemployable | demographics |  |  |  | Y |
| VAPensionFlag | VA Pension | demographics |  |  |  | Y |
| charlson_index | Charlson comorbidity index | comorbidities |  |  |  |  |
| charlson_indexf | Charlson comorbidity index range | comorbidities |  |  |  |  |
| 1_CHARL_MYOCARDIAL_INFARCTION | Myocardial Infarction | comorbidities |  |  |  |  |
| 2_CHARL_CONGESTIVE_HEART_FAILURE | Congestive Heart Failure | comorbidities |  |  |  |  |
| 3_CHARL_PERIPHRAL_VASCULAR_DISEASE | Peripheral Vascular Disease | comorbidities |  |  |  |  |
| 4_CHARL_CEREBROVASCULAR_DISEASE | Cerebrovascular Disease | comorbidities |  |  |  |  |
| 5_CHARL_DEMENTIA | Dementia | comorbidities |  |  |  |  |
| 6_CHARL_CHRONIC_PULMONARY_DISEASE | Chronic Pulmonary Disease | comorbidities |  |  |  |  |
| 7_CHARL_CONNECTIVE_TISSUE_RHEUMATIC_DISEASE | Connective Tissue Rheumatic Disease | comorbidities |  |  |  |  |
| 8_CHARL_PEPTIC_ULCER_DISEASE | Peptic Ulcer Disease | comorbidities |  |  |  |  |
| 9_CHARL_MILD_LIVER_DISEASE | Mild Liver Disease | comorbidities |  |  |  |  |
| 10_CHARL_DIABETES_WITHOUT_COMPLICATES | Uncomplicated Diabetes | comorbidities |  |  |  |  |
| 11_CHARL_DIABETES_WITH_COMPLICATES | Complicated Diabetes | comorbidities |  |  |  |  |
| 12_CHARL_PARAPLEGIA_AND_HEMIPLEGIA | Paraplegia and Hemiplegia | comorbidities |  |  |  |  |
| 13_CHARL_RENAL_DISEASE | Renal Disease | comorbidities |  |  |  |  |
| 14_CHARL_CANCER | Cancer | comorbidities |  |  |  |  |
| 15_CHARL_MOD_SEVERE_LIVER_DISEASE | Severe Liver Disease | comorbidities |  |  |  |  |
| 16_CHARL_METASTATIC_CARCINOMA | Metastatic Carcinoma | comorbidities |  |  |  |  |
| 17_CHARL_AIDS_HIV | HIV/AIDS | comorbidities |  |  |  |  |
| emergency_care | Emergency care visits | utilization |  |  |  |  |
| cardiology | Cardiology visits | utilization |  |  |  |  |
| oncology | Oncology visits | utilization |  |  |  |  |
| radiology | Radiology visits | utilization |  |  |  |  |
| mental_health | Mental health visits | utilization |  |  |  |  |
| other_nonface | Non-face-to-face visits | utilization |  |  |  | Y |
| primarycare | Primary care visits | utilization |  |  |  |  |
| phone_care | Phone visits | utilization |  |  |  |  |
| pc_phone_care | Primary care phone visits | utilization |  |  |  |  |
| renal | Renal visits | utilization |  |  |  |  |
| surg | Surgical visits | utilization |  |  |  |  |
| dental | Dental visits | utilization |  |  |  |  |
| CT | CT scan visits | utilization |  |  |  |  |
| MRI | MRI visits | utilization |  |  |  |  |
| diagnositic_radiology | Diagnostic radiology visits | utilization |  |  |  |  |
| echo | Echocardiogram visits | utilization |  |  |  |  |
| nuclear | Nuclear radiology visits | utilization |  |  |  |  |
| ultrasound | Ultrasound visits | utilization |  |  |  |  |
| xray | X-ray visits | utilization |  |  |  |  |
| inpt_surgery | Inpatient surgery visit | utilization |  |  |  |  |
| inpt_cardiology | Inpatient cardiology visit | utilization |  |  |  |  |
| inpt_medical | Inpatient medical visit | utilization |  |  |  |  |
| inpt_nursing_rehab | Inpatient nursing rehab visit | utilization |  |  |  |  |
| inpt_psychiatric | Inpatient psychiatric visit | utilization |  |  |  |  |
| inpt_hospice | Inpatient hospice visit | utilization |  |  |  |  |
| inpt_spinal_cord | Inpatient spinal cord visit | utilization |  |  |  |  |
| inpt_neurology_non_sc | Inpatient non-spinal cord neurology visit | utilization |  |  |  |  |
| inpt_oncology | Inpatient oncology visit | utilization |  |  |  |  |
| inpt_substanceuse | Inpatient substance use visit | utilization |  |  |  |  |
| inpt_grptotind | Inpatient visit | utilization |  |  |  |  |
| inpt_grptotsum | inpatient visit group total | utilization |  |  |  |  |
| inpt_days | Total inpatient visits | utilization |  |  |  |  |
| out_card | Outpatient cardiology visit | utilization |  |  |  |  |
| out_dental | Outpatient dental visit | utilization |  |  |  |  |
| out_er | Outpatient ER visit | utilization |  |  |  |  |
| out_neuro | Outpatient Neurology visit | utilization |  |  |  |  |
| out_nonface | Outpatient non-face-to-face visit | utilization |  |  |  | Y |
| out_onc | Outpatient oncology visit | utilization |  |  |  |  |
| out_pc | Outpatient primary care visit | utilization |  |  |  |  |
| out_pcphonecare | Outpatient primary care phone visit | utilization |  |  |  |  |
| out_phonecare | Outpatient phone visit | utilization |  |  |  |  |
| out_psychiatric | Outpatient psychiatry visit | utilization |  |  |  |  |
| out_rad | Outpatient radiology visit | utilization |  |  |  |  |
| out_renal | Outpatient nephrology visit | utilization |  |  |  |  |
| out_substanceuse | Outpatient substance use visit | utilization |  |  |  |  |
| out_surg | Outpatinet surgery visit | utilization |  |  |  |  |
| out_CT | Outpatient CT visit | utilization |  |  |  |  |
| out_MRI | Outpatient MRI visit | utilization |  |  |  |  |
| out_diagrad | Outpatient diagnostic radiology visit | utilization |  |  |  |  |
| out_echo | Outpatinet echocardiography visit | utilization |  |  |  |  |
| out_nuclear | Outpatient nuclear radiology visit | utilization |  |  |  |  |
| out_ultrasound | Outpatient ultrasound visit | utilization |  |  |  |  |
| out_xray | Outpatient X-ray visit | utilization |  |  |  |  |
| out_diagrad_sum | Outpatient diagnostic radiology visits | utilization |  |  |  |  |
| out_sum | Outpatient total visits | utilization |  |  |  |  |
| ALT_n | # of ALT | laboratories |  |  |  |  |
| ALT_mean | ALT mean | laboratories |  |  |  |  |
| ALT_std | ALT standard deviation | laboratories |  |  |  |  |
| ALT_min | ALT minimum | laboratories |  |  |  |  |
| ALT_max | ALT maximum | laboratories |  |  |  |  |
| ALBUMIN_n | # of Albumin | laboratories |  |  |  |  |
| ALBUMIN_mean | Albumin mean | laboratories |  |  |  |  |
| ALBUMIN_std | Albumin standard deviation | laboratories |  |  |  |  |
| ALBUMIN_min | Albumin minimum | laboratories |  |  |  |  |
| ALBUMIN_max | Albumin maximum | laboratories |  |  |  |  |
| ALKALINE_PHOSPHATASE_n | # of Alkaline Phosphatase | laboratories |  |  |  |  |
| ALKALINE_PHOSPHATASE_mean | Alkaline Phosphatase mean | laboratories |  |  |  |  |
| ALKALINE_PHOSPHATASE_std | Alkaline Phosphatase standard deviation | laboratories |  |  |  |  |
| ALKALINE_PHOSPHATASE_min | Alkaline Phosphatase minimum | laboratories |  |  |  |  |
| ALKALINE_PHOSPHATASE_max | Alkaline Phosphatase maximum | laboratories |  |  |  |  |
| AST_n | # of AST | laboratories |  |  |  |  |
| AST_mean | AST mean | laboratories |  |  |  |  |
| AST_std | AST standard deviation | laboratories |  |  |  |  |
| AST_min | AST minimum | laboratories |  |  |  |  |
| AST_max | AST maximum | laboratories |  |  |  |  |
| BICARB_n | # of Bicarbonate | laboratories |  |  |  |  |
| BICARB_mean | Bicarbonate mean | laboratories |  |  |  |  |
| BICARB_median | Bicarbonate median | laboratories |  |  |  |  |
| BICARB_std | Bicarbonate standard deviation | laboratories |  |  |  |  |
| BICARB_min | Bicarbonate minimum | laboratories |  |  |  |  |
| BICARB_max | Bicarbonate maximum | laboratories |  |  |  |  |
| BILIRUBIN_n | # of Total Bilirubin | laboratories |  |  |  |  |
| BILIRUBIN_mean | Total Bilirubin mean | laboratories |  |  |  |  |
| BILIRUBIN_std | Total Bilirubin standard deviation | laboratories |  |  |  |  |
| BILIRUBIN_min | Total Bilirubin minimum | laboratories |  |  |  |  |
| BILIRUBIN_max | Total Bilirubin maximum | laboratories |  |  |  |  |
| C_REACTIVE_n | # of C reactive protein | laboratories |  |  |  |  |
| C_REACTIVE_mean | C reactive protein mean | laboratories |  |  |  |  |
| C_REACTIVE_median | C reactive protein median | laboratories |  |  |  |  |
| C_REACTIVE_std | C reactive protein standard deviation | laboratories |  |  |  |  |
| C_REACTIVE_min | C reactive protein minimum | laboratories |  |  |  |  |
| C_REACTIVE_max | C reactive protein maximum | laboratories |  |  |  |  |
| CHOLESTEROL_n | # of Cholesterol | laboratories |  |  |  |  |
| CHOLESTEROL_mean | Cholesterol mean | laboratories |  |  |  |  |
| CHOLESTEROL_median | Cholesterol median | laboratories |  |  |  |  |
| CHOLESTEROL_std | Cholesterol standard deviation | laboratories |  |  |  |  |
| CHOLESTEROL_min | Cholesterol minimum | laboratories |  |  |  |  |
| CHOLESTEROL_max | Cholesterol maximum | laboratories |  |  |  |  |
| CHOLESTEROL_LDL_n | # of LDL cholesterol | laboratories |  |  |  |  |
| CHOLESTEROL_LDL_mean | LDL cholesterol mean | laboratories |  |  |  |  |
| CHOLESTEROL_LDL_std | LDL cholesterol standard deviation | laboratories |  |  |  |  |
| CHOLESTEROL_LDL_min | LDL cholesterol minimum | laboratories |  |  |  |  |
| CHOLESTEROL_LDL_max | LDL cholesterol maximum | laboratories |  |  |  |  |
| CREATININE_n | # of Creatinine | laboratories |  |  |  |  |
| CREATININE_mean | Creatinine mean | laboratories |  |  |  |  |
| CREATININE_std | Creatinine standard deviation | laboratories |  |  |  |  |
| CREATININE_min | Creatinine minimum | laboratories |  |  |  |  |
| CREATININE_max | Creatinine maximum | laboratories |  |  |  |  |
| GLUCOSE_n | # of Glucose | laboratories |  |  |  |  |
| GLUCOSE_mean | Glucose mean | laboratories |  |  |  |  |
| GLUCOSE_std | Glucose standard deviation | laboratories |  |  |  |  |
| GLUCOSE_min | Glucose minimum | laboratories |  |  |  |  |
| GLUCOSE_max | Glucose maximum | laboratories |  |  |  |  |
| HEMOGLOBIN_n | # of Hemoglobin | laboratories |  |  |  |  |
| HEMOGLOBIN_mean | Hemoglobin mean | laboratories |  |  |  |  |
| HEMOGLOBIN_std | Hemoglobin standard deviation | laboratories |  |  |  |  |
| HEMOGLOBIN_min | Hemoglobin minimum | laboratories |  |  |  |  |
| HEMOGLOBIN_max | Hemoglobin maximum | laboratories |  |  |  |  |
| A1C_TOTAL_n | # of Hemoglobin A1C | laboratories |  |  |  |  |
| A1C_TOTAL_mean | Hemoglobin A1C mean | laboratories |  |  |  |  |
| A1C_TOTAL_std | Hemoglobin A1C standard deviation | laboratories |  |  |  |  |
| A1C_TOTAL_min | Hemoglobin A1C minimum | laboratories |  |  |  |  |
| A1C_TOTAL_max | Hemoglobin A1C maximum | laboratories |  |  |  |  |
| INR_n | # of INR | laboratories |  |  |  |  |
| INR_mean | INR mean | laboratories |  |  |  |  |
| INR_median | INR median | laboratories |  |  |  |  |
| INR_std | INR standard deviation | laboratories |  |  |  |  |
| INR_min | INR minimum | laboratories |  |  |  |  |
| INR_max | INR maximum | laboratories |  |  |  |  |
| LACTATE_n | # of Lactate | laboratories |  |  |  |  |
| LACTATE_mean | Lactate mean | laboratories |  |  |  |  |
| LACTATE_median | Lactate median | laboratories |  |  |  |  |
| LACTATE_std | Lactate standard deviation | laboratories |  |  |  |  |
| LACTATE_min | Lactate minimum | laboratories |  |  |  |  |
| LACTATE_max | Lactate maximum | laboratories |  |  |  |  |
| LEUKOCYTES_n | # of Leukocytes | laboratories |  |  |  |  |
| LEUKOCYTES_mean | Leukocytes mean | laboratories |  |  |  |  |
| LEUKOCYTES_std | Leukocytes standard deviation | laboratories |  |  |  |  |
| LEUKOCYTES_min | Leukocytes minimum | laboratories |  |  |  |  |
| LEUKOCYTES_max | Leukocytes maximum | laboratories |  |  |  |  |
| MCV_n | # of MCV | laboratories |  |  |  |  |
| MCV_mean | MCV mean | laboratories |  |  |  |  |
| MCV_median | MCV median | laboratories |  |  |  |  |
| MCV_std | MCV standard deviation | laboratories |  |  |  |  |
| MCV_min | MCV minimum | laboratories |  |  |  |  |
| MCV_max | MCV maximum | laboratories |  |  |  |  |
| PLATELET_n | # of Platelet | laboratories |  |  |  |  |
| PLATELET_mean | Platelet mean | laboratories |  |  |  |  |
| PLATELET_std | Platelet standard deviation | laboratories |  |  |  |  |
| PLATELET_min | Platelet minimum | laboratories |  |  |  |  |
| PLATELET_max | Platelet maximum | laboratories |  |  |  |  |
| POTASSIUM_n | # of Potassium | laboratories |  |  |  |  |
| POTASSIUM_mean | Potassium mean | laboratories |  |  |  |  |
| POTASSIUM_median | Potassium median | laboratories |  |  |  |  |
| POTASSIUM_std | Potassium standard deviation | laboratories |  |  |  |  |
| POTASSIUM_min | Potassium minimum | laboratories |  |  |  |  |
| POTASSIUM_max | Potassium maximum | laboratories |  |  |  |  |
| SODIUM_n | # of Sodium | laboratories |  |  |  |  |
| SODIUM_mean | Sodium mean | laboratories |  |  |  |  |
| SODIUM_std | Sodium standard deviation | laboratories |  |  |  |  |
| SODIUM_min | Sodium minimum | laboratories |  |  |  |  |
| SODIUM_max | Sodium maximum | laboratories |  |  |  |  |
| UREA_NITROGEN_n | # of Urea Nitrogen | laboratories |  |  |  |  |
| UREA_NITROGEN_mean | Urea Nitrogen mean | laboratories |  |  |  |  |
| UREA_NITROGEN_std | Urea Nitrogen standard deviation | laboratories |  |  |  |  |
| UREA_NITROGEN_min | Urea Nitrogen minimum | laboratories |  |  |  |  |
| UREA_NITROGEN_max | Urea Nitrogen maximum | laboratories |  |  |  |  |
| BMI_n | BMI measurements | vital signs |  |  |  |  |
| BMI | BMI group | vital signs |  |  |  |  |
| BMI_mean | Mean BMI | vital signs |  |  |  |  |
| HEIGHT_n | Height measurements | vital signs |  |  |  |  |
| HEIGHT | Height group | vital signs |  |  |  |  |
| HEIGHT_std | Height standard deviation | vital signs |  |  |  |  |
| HEIGHT_min | Height min | vital signs |  |  |  |  |
| HEIGHT_max | Height max | vital signs |  |  |  |  |
| HEIGHT_variance | Height variance | vital signs |  |  |  |  |
| WEIGHT_n | Weight measurements | vital signs |  |  |  |  |
| WEIGHT | Weight group | vital signs |  |  |  |  |
| WEIGHT_std | Weight standard deviation | vital signs |  |  |  |  |
| WEIGHT_min | Weight min | vital signs |  |  |  |  |
| WEIGHT_max | Weight max | vital signs |  |  |  |  |
| WEIGHT_variance | Weight variance | vital signs |  |  |  |  |
| PULSE_n | Heart rate measurements | vital signs |  |  |  |  |
| PULSE | Heart rate group | vital signs |  |  |  |  |
| PULSE_mean | Heart rate mean | vital signs |  |  |  |  |
| PULSE_std | Heart rate standard deviation | vital signs |  |  |  |  |
| PULSE_min | Heart rate min | vital signs |  |  |  |  |
| PULSE_max | Heart rate max | vital signs |  |  |  |  |
| PULSE_variance | Heart rate variance | vital signs |  |  |  |  |
| PULSE_OXIMETRY_n | Pulse oximetry measurements | vital signs |  |  |  |  |
| PULSE_OXIMETRY | Pulse oximetry group | vital signs |  |  |  |  |
| PULSE_OXIMETRY_mean | Pulse oximetry mean | vital signs |  |  |  |  |
| PULSE_OXIMETRY_std | Pulse oximetry standard deviation | vital signs |  |  |  |  |
| PULSE_OXIMETRY_min | Pulse oximetry min | vital signs |  |  |  |  |
| PULSE_OXIMETRY_max | Pulse oximetry max | vital signs |  |  |  |  |
| PULSE_OXIMETRY_variance | Pulse oximetry variance | vital signs |  |  |  |  |
| RESPIRATION_n | Respiratory rate measurements | vital signs |  |  |  |  |
| RESPIRATION | Respiratory rate group | vital signs |  |  |  |  |
| RESPIRATION_mean | Respiratory rate mean | vital signs |  |  |  |  |
| RESPIRATION_std | Respiratory rate standard deviation | vital signs |  |  |  |  |
| RESPIRATION_min | Respiratory rate min | vital signs |  |  |  |  |
| RESPIRATION_max | Respiratory rate max | vital signs |  |  |  |  |
| RESPIRATION_variance | Respiratory rate variance | vital signs |  |  |  |  |
| DIASTOLIC_n | Diastolic BP measurements | vital signs |  |  |  |  |
| DIASTOLIC | Diastolic BP group | vital signs |  |  |  |  |
| DIASTOLIC_mean | Diastolic BP mean | vital signs |  |  |  |  |
| DIASTOLIC_std | Diastolic BP standard deviation | vital signs |  |  |  |  |
| DIASTOLIC_min | Diastolic BP min | vital signs |  |  |  |  |
| DIASTOLIC_max | Diastolic BP max | vital signs |  |  |  |  |
| DIASTOLIC_variance | Diastolic BP variance | vital signs |  |  |  |  |
| SYSTOLIC_n | Systolic BP measurements | vital signs |  |  |  |  |
| SYSTOLIC | Systolic BP group | vital signs |  |  |  |  |
| SYSTOLIC_mean | Systolic BP mean | vital signs |  |  |  |  |
| SYSTOLIC_std | Systolic BP standard deviation | vital signs |  |  |  |  |
| SYSTOLIC_min | Systolic BP min | vital signs |  |  |  |  |
| SYSTOLIC_max | Systolic BP max | vital signs |  |  |  |  |
| SYSTOLIC_variance | Systolic BP variance | vital signs |  |  |  |  |
| CENTRAL_VENOUS_PRESSURE_n | Central venous pressure measurements | vital signs |  |  |  |  |
| CENTRAL_VENOUS_PRESSURE | Central venous pressure group | vital signs |  |  |  |  |
| CENTRAL_VENOUS_PRESSURE_median | Central venous pressure mean | vital signs |  |  |  |  |
| CENTRAL_VENOUS_PRESSURE_mean | Central venous pressure median | vital signs |  |  |  |  |
| CENTRAL_VENOUS_PRESSURE_std | Central venous pressure standard deviation | vital signs |  |  |  |  |
| CENTRAL_VENOUS_PRESSURE_min | Central venous pressure min | vital signs |  |  |  |  |
| CENTRAL_VENOUS_PRESSURE_max | Central venous pressure max | vital signs |  |  |  |  |
| CENTRAL_VENOUS_PRESSURE_variance | Central venous pressure variance | vital signs |  |  |  |  |
| PAIN_n | Pain score measurements | vital signs |  |  |  |  |
| PAIN | Pain score group | vital signs |  |  |  |  |
| PAIN_mean | Pain score mean | vital signs |  |  |  |  |
| PAIN_std | Pain score standard deviation | vital signs |  |  |  |  |
| PAIN_min | Pain score min | vital signs |  |  |  |  |
| PAIN_max | Pain score max | vital signs |  |  |  |  |
| PAIN_variance | Pain score variance | vital signs |  |  |  |  |
| CIRCUMFERENCE_GIRTH_n | Circumference girth measurements | vital signs |  |  |  |  |
| CIRCUMFERENCE_GIRTH | Circumference girth group | vital signs |  |  |  |  |
| CIRCUMFERENCE_GIRTH_median | Circumference girth median | vital signs |  |  |  |  |
| CIRCUMFERENCE_GIRTH_std | Circumference girth standard deviation | vital signs |  |  |  |  |
| CIRCUMFERENCE_GIRTH_min | Circumference girth min | vital signs |  |  |  |  |
| CIRCUMFERENCE_GIRTH_max | Circumference girth max | vital signs |  |  |  |  |
| CIRCUMFERENCE_GIRTH_variance | Circumference girth variance | vital signs |  |  |  |  |
| TEMPERATURE_n | Temperature measurements | vital signs |  |  |  |  |
| TEMPERATURE | Temperature group | vital signs |  |  |  |  |
| TEMPERATURE_std | Temperature standard deviation | vital signs |  |  |  |  |
| TEMPERATURE_min | Temperature min | vital signs |  |  |  |  |
| TEMPERATURE_max | Temperature max | vital signs |  |  |  |  |
| TEMPERATURE_variance | Temperature variance | vital signs |  |  |  |  |
