# Supplementary material for: A machine learning approach to identify distinct subgroups of veterans at risk for hospitalization or death using administrative and electronic health record data
Source: PLoS One. 2021 Feb 19;16(2):e0247203. doi: 10.1371/journal.pone.0247203 (PMC7894856; doi:10.1371/journal.pone.0247203)
Supplement: S2 Table — (DOCX) [file pone.0247203.s002.docx]

**S2 Table. Characteristics and outcomes of clusters**

View on https://github.com/Parikh-Ravi/Parikh_Phenotyping.git
