# Supplementary material for: A machine learning approach to identify distinct subgroups of veterans at risk for hospitalization or death using administrative and electronic health record data
Source: PLoS One. 2021 Feb 19;16(2):e0247203. doi: 10.1371/journal.pone.0247203 (PMC7894856; doi:10.1371/journal.pone.0247203)
Supplement: S3 Table — (DOCX) [file pone.0247203.s003.docx]

**S3 Table. Comparison of 5 most important variables that contribute to clusters within validation cohort**

| Cluster | #1 Variable | #2 Variable | #3 Variable | #4 Variable | #5 Variable |
| --- | --- | --- | --- | --- | --- |
| Low comorbidity burden | gender | # of non-insulin glucose lowering agents | Uncomplicated Diabetes | # of insulins | Rheumatologic Disease |
| Insulin-dependent diabetes | Uncomplicated Diabetes | # of non-insulin glucose lowering agents | Hemoglobin A1C median | Glucose median | Hypothyroidism |
| Psychoses without drug abuse | Psychoses | # of antipsychotics | Outpatient psychiatric visits | Inpatient psychiatric visits | Height median |
| Chronic renal disease | Renal Disease | Creatinine median | Urea Nitrogen median | Complicated Hypertension | Hemoglobin median |
| Ischemic heart disease | Outpatient cardiology visits | Outpatient echocardiography visits | Outpatient nuclear radiology visits | # of beta blockers | # of antiarrhythmics |
| High Missingness | Number of missing variables | Uncomplicated Hypertension | Outpatient primary care visits | # of lipid modifying agents | Tobacco Use |
| Uncomplicated surgery | Inpatient surgery visits | Outpatient surgery visits | Hemoglobin median | # of opioids | Outpatient X-ray visits |
| Valvular heart disease | Valvular Disease | Age as of 1/1/2014 | Outpatient cardiology visits | # of antithrombotics | Congestive Heart Failure |
| Pulmonary vascular disease | Pulmonary Circulation Disease | # of antithrombotics | Coagulopathy | Pulse oximetry median | Chronic Pulmonary Disease |
| Chronic liver disease | AST median | ALT median | Liver Disease | Alkaline Phosphatase median | Alcohol Abuse |
| Hispanics predominant | ethnicity | gender | Albumin median | Hypothyroidism | # of anti-inflammatory |
| Iron-deficiency anemia | Deficiency Anemia | Leukocytes median | Age as of 1/1/2014 | # of insulins | Hemoglobin median |
| Cardiac arrhythmias | # of antiarrhythmics | Cardiac Arrhythmia | Outpatient cardiology visits | Outpatient echocardiography visits | # of antithrombotics |
| Thyroid disease with diabetes | Hypothyroidism | # of thyroid agents | Uncomplicated Diabetes | # of non-insulin glucose lowering agents | Hemoglobin A1C median |
| Thyroid disease without diabetes | Hypothyroidism | # of thyroid agents | # of non-insulin glucose lowering agents | Uncomplicated Diabetes | gender |
| Polysubstance use - not otherwise specified | Drug Abuse | Cannabis Use | Cocaine Use | Alcohol Abuse | HIV/AIDS |
| Polysubstance use - opioid predominant | Opiod Use | Drug Abuse | Outpatient substance use visits | # of opioid abuse therapies | Age as of 1/1/2014 |
| Females predominant | gender | Height median | Age as of 1/1/2014 | # of non-insulin glucose lowering agents | Hypothyroidism |
| Polysubstance use - sedative predominant | Sedative Use | Drug Abuse | Amphetamine Use | Opiod Use | Inpatient psychiatric visits |
| Metastatic cancer | Metastatic Solid Tumors | Outpatient oncology visits | Non-metastatic Solid Tumors | Outpatient CT visits | Outpatient nuclear radiology visits |
| Polysubstance use - amphetamine predominant | Amphetamine Use | Drug Abuse | Sedative Use | Outpatient substance use visits | Cannabis Use |
| Paralysis/spinal cord injuries | Paralysis | Inpatient spinal cord visits | Outpatient neurology visits | Outpatient phone visits | Systolic BP median |
| Blood-loss anemia | Blood Loss Anemia | Deficiency Anemia | Hemoglobin median | Outpatient non-face-to-face visits | Gastroparesis |
| Rheumatologic disease | Rheumatologic Disease | Lymphoma | Outpatient primary care phone visits | Metastatic Solid Tumors | Chronic Pulmonary Disease |
| Home-based care | Outpatient non-face-to-face visits | Outpatient primary care phone visits | Age as of 1/1/2014 | CANscore 2014 | Outpatient primary care visits |
| Lymphoma without inpatient utilization | Lymphoma | Outpatient oncology visits | Medicaid Beneficiary | Peptic Ulcer Disease | Outpatient non-face-to-face visits |
| Peptic ulcer disease | Peptic Ulcer Disease | Gastroparesis | Blood Loss Anemia | Outpatient primary care phone visits | # of antiinfectives |
| Medicaid predominant | Medicaid Beneficiary | HIV/AIDS | # of antiinfectives | Service Connected Percentage | Amphetamine Use |
| HIV/AIDS | HIV/AIDS | Medicaid Beneficiary | Age as of 1/1/2014 | race group | Leukocytes median |
| Post-surgical infection | # of antiinfectives | Outpatient surgery visits | Outpatient primary care visits | Outpatient primary care phone visits | Rheumatologic Disease |
