# Supplementary material for: A machine learning approach to identify distinct subgroups of veterans at risk for hospitalization or death using administrative and electronic health record data
Source: PLoS One. 2021 Feb 19;16(2):e0247203. doi: 10.1371/journal.pone.0247203 (PMC7894856; doi:10.1371/journal.pone.0247203)
Supplement: S4 Table — (DOCX) [file pone.0247203.s004.docx]

**S4 Table. Outcomes (2015-2016) by cluster**

| **Category** | **Cluster Label** | **Sample size** | **Denominator for utilization (sample size - death)** | **Hospitalization (n, %)** | **Hospital days (median, IQR)** | **Emergency room utilization (n, %)** | **Number of ED visits (median,IQR)** | **Died (n, %)** |
| --- | --- | --- | --- | --- | --- | --- | --- | --- |
| **Comorbidity** | Low comorbidity burden | 2446 | 2199 | 511 (23.2) | 0.00 [0.00, 0.00] | 999 (45.4) | 0.00 [0.00, 2.00] | 247 (10.1) |
| **Comorbidity** | Insulin-dependent diabetes | 1156 | 1068 | 252 (23.6) | 0.00 [0.00, 0.00] | 498 (46.6) | 0.00 [0.00, 2.00] | 88 (7.6) |
| **Psychobehavioral** | Psychoses without drug abuse | 150 | 144 | 57 (39.6) | 0.00 [0.00, 12.00] | 73 (50.7) | 1.00 [0.00, 2.25] | 6 (4.0) |
| **Comorbidity** | Chronic renal disease | 390 | 312 | 81 (26.0) | 0.00 [0.00, 1.00] | 123 (39.4) | 0.00 [0.00, 1.00] | 78 (20.0) |
| **Comorbidity** | Ischemic heart disease | 50 | 47 | 27 (57.4) | 2.00 [0.00, 6.00] | 28 (59.6) | 1.00 [0.00, 2.00] | 3 (6.0) |
| **Comorbidity** | High Missingness | 249 | 181 | 19 (10.5) | 0.00 [0.00, 0.00] | 51 (28.2) | 0.00 [0.00, 1.00] | 68 (27.3) |
| **Comorbidity** | Uncomplicated surgery | 84 | 74 | 73 (98.6) | 14.00 [8.00, 30.00] | 41 (55.4) | 1.00 [0.00, 2.00] | 10 (11.9) |
| **Comorbidity** | Valvular heart disease | 136 | 109 | 43 (39.4) | 0.00 [0.00, 6.00] | 53 (48.6) | 0.00 [0.00, 2.00] | 27 (19.9) |
| **Comorbidity** | Pulmonary vascular disease | 65 | 56 | 27 (48.2) | 0.00 [0.00, 6.00] | 36 (64.3) | 1.00 [0.00, 3.00] | 9 (13.8) |
| **Comorbidity** | Chronic liver disease | 157 | 134 | 45 (33.6) | 0.00 [0.00, 3.50] | 81 (60.4) | 1.00 [0.00, 3.00] | 23 (14.6) |
| **Sociodemographic** | Hispanics predominant | 154 | 147 | 29 (19.7) | 0.00 [0.00, 0.00] | 78 (53.1) | 1.00 [0.00, 2.00] | 7 (4.5) |
| **Comorbidity** | Iron-deficiency anemia | 70 | 60 | 19 (31.7) | 0.00 [0.00, 2.00] | 23 (38.3) | 0.00 [0.00, 1.00] | 10 (14.3) |
| **Comorbidity** | Cardiac arrhythmias | 115 | 101 | 45 (44.6) | 0.00 [0.00, 6.00] | 44 (43.6) | 0.00 [0.00, 2.00] | 14 (12.2) |
| **Comorbidity** | Thyroid disease with diabetes | 115 | 109 | 28 (25.7) | 0.00 [0.00, 1.00] | 51 (46.8) | 0.00 [0.00, 2.00] | 6 (5.2) |
| **Comorbidity** | Thyroid disease without diabetes | 190 | 170 | 44 (25.9) | 0.00 [0.00, 1.00] | 78 (45.9) | 0.00 [0.00, 2.00] | 20 (10.5) |
| **Psychobehavioral** | Polysubstance use - not otherwise specified | 273 | 260 | 128 (49.2) | 0.00 [0.00, 12.50] | 163 (62.7) | 1.00 [0.00, 3.00] | 13 (4.8) |
| **Psychobehavioral** | Polysubstance use - opioid predominant | 116 | 111 | 41 (36.9) | 0.00 [0.00, 6.00] | 70 (63.1) | 1.00 [0.00, 3.00] | 5 (4.3) |
| **Sociodemographic** | Females predominant | 320 | 317 | 60 (18.9) | 0.00 [0.00, 0.00] | 191 (60.3) | 1.00 [0.00, 3.00] | 3 (0.9) |
| **Psychobehavioral** | Polysubstance use - sedative predominant | 50 | 47 | 24 (51.1) | 1.00 [0.00, 13.00] | 33 (70.2) | 3.00 [0.00, 6.00] | 3 (6.0) |
| **Comorbidity** | Metastatic cancer | 128 | 80 | 37 (46.2) | 0.00 [0.00, 7.25] | 30 (37.5) | 0.00 [0.00, 2.00] | 48 (37.5) |
| **Psychobehavioral** | Polysubstance use - amphetamine predominant | 65 | 59 | 32 (54.2) | 2.00 [0.00, 13.00] | 34 (57.6) | 1.00 [0.00, 3.00] | 6 (9.2) |
| **Comorbidity** | Paralysis/spinal cord injuries | 64 | 54 | 31 (57.4) | 4.50 [0.00, 21.75] | 20 (37.0) | 0.00 [0.00, 1.00] | 10 (15.6) |
| **Comorbidity** | Blood-loss anemia | 54 | 43 | 32 (74.4) | 6.00 [1.00, 15.00] | 33 (76.7) | 1.00 [1.00, 3.00] | 11 (20.4) |
| **Comorbidity** | Rheumatologic disease | 213 | 197 | 65 (33.0) | 0.00 [0.00, 2.00] | 96 (48.7) | 0.00 [0.00, 3.00] | 16 (7.5) |
| **Sociodemographic** | Home-based care | 136 | 74 | 33 (44.6) | 0.00 [0.00, 12.75] | 26 (35.1) | 0.00 [0.00, 1.75] | 62 (45.6) |
| **Comorbidity** | Lymphoma without inpatient utilization | 103 | 74 | 24 (32.4) | 0.00 [0.00, 2.00] | 31 (41.9) | 0.00 [0.00, 1.00] | 29 (28.2) |
| **Comorbidity** | Peptic ulcer disease | 119 | 111 | 46 (41.4) | 0.00 [0.00, 4.50] | 56 (50.5) | 1.00 [0.00, 2.00] | 8 (6.7) |
| **Sociodemographic** | Medicaid predominant | 117 | 102 | 37 (36.3) | 0.00 [0.00, 4.75] | 46 (45.1) | 0.00 [0.00, 2.00] | 15 (12.8) |
| **Comorbidity** | HIV/AIDS | 108 | 103 | 38 (36.9) | 0.00 [0.00, 4.00] | 48 (46.6) | 0.00 [0.00, 3.00] | 5 (4.6) |
| **Comorbidity** | Post-surgical infection | 91 | 77 | 26 (33.8) | 0.00 [0.00, 2.00] | 45 (58.4) | 1.00 [0.00, 3.00] | 14 (15.4) |
