# Supplementary figures and images for: A machine learning approach to identify distinct subgroups of veterans at risk for hospitalization or death using administrative and electronic health record data
Source: PLoS One. 2021 Feb 19;16(2):e0247203. doi: 10.1371/journal.pone.0247203 (PMC7894856; doi:10.1371/journal.pone.0247203)

**S1 Fig. Distribution of CAN scores in cohort**


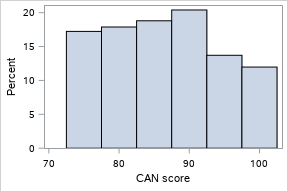

Supplement: S1 Fig — (DOCX) [file pone.0247203.s005.docx]
