# Supplementary material for: A machine learning approach to identify distinct subgroups of veterans at risk for hospitalization or death using administrative and electronic health record data
Source: PLoS One. 2021 Feb 19;16(2):e0247203. doi: 10.1371/journal.pone.0247203 (PMC7894856; doi:10.1371/journal.pone.0247203)
Supplement: S1 File — (DOCX) [file pone.0247203.s006.docx]

# Methods

## List of Features

We extracted 415 features from the VA Corporate Data Warehouse (CDW) between January 1 and December 31, 2014. Features were broadly grouped into the following six categories: demographics, comorbidities, pharmacy, vital signs, laboratories, and prior utilization. Laboratories and vital signs could be measured for more than once per year. After reviewing descriptive statistics and trends in laboratories and vital signs, median values of these variables were chosen to summarize multiple values within the year to reduce noise. We first selected features based on clinical relevance and data availability. Pharmacy records were summarized into two measures: (1) polypharmacy: number of unique medications per therapeutic class; and (2) adherence: weighted proportion of days covered. We then removed variables with extremely low variance based on two criteria: (1) the ratio of the number of observations with the second most common value to the number of observations with the most common value was ≥ 0.99; and (2) the percentage of unique values (the number of unique values divided by the number of observations) was ≤ 1%. We also calculated Pearson’s correlation coefficient among all pairs of continuous variables. For any pair with a correlation greater than 0.80, we removed one of the two variables, based on clinical input from the first-author (RP, an internal medicine physician and oncology specialist) and senior author (AN, an internal medicine physician). After this reduced, high-quality set of variables was constructed, we calculated the number of missing values for each subject and included this as an additional feature. This pre-processing step resulted in 119 variables that are used in the subsequent analysis.

## Missing Imputation

Among the 119 features selected, 24 (20%) contained various amounts of missing values (range: 3-35% within each variable). Variables with missing values were broadly grouped into laboratory values (n=14), vital signs (n=9), and sociodemographic (n=1) variables. We assumed that data was missing at random.

We chose to use the Fully Conditional Specification (FCS, or chained equations) method to impute missing data to reduce bias, rather than performing a complete case analysis. Our rationale for imputation was that missing information was informative in a real-world dataset and thus we wanted to impute values to increase power but also identify missingness for the purpose of clustering. For a data set with an arbitrary missing data pattern, FCS assumes the existence of a joint distribution for these variables (Brand 1999; Van Buuren 2007). The FCS method involves two phases in each imputation: the preliminary filled-in phase followed by the imputation phase. Missing data was imputed once using FCS in order to enable calculation of pairwise distance measures between all observations in the data.

## Dimension Reduction

When working with datasets with large numbers of variables, dimension reduction is often employed with the goal of reducing the number of features while retaining as much of the original information in the data as possible. We utilized a non-linear dimension reduction algorithm known as t-distributed stochastic neighbor embedding (t-SNE) to create a low-dimension representation of the dataset, which has been demonstrated to work well in prior work. Specifically, we ran the Barnes-Hut implementation of t-SNE and retained the first two dimensions in the reduced space in order to facilitate visualization. We ran t-SNE for 5000 iterations with the perplexity parameter of 50 and the default speed/accuracy trade-off parameter (theta) of 0.5. The algorithm returns a solution, i.e., location of observations in the lower-dimensional space, that minimizes the Kullback-Leibler divergence between the distribution of pairwise similarities in the original space and the distribution of pairwise similarities in the lower-dimensional space, where similarity is measured using Gaussian and Student’s t-distribution kernels, respectively.

We also applied Principal Component Analysis to reduce dimensions instead of t-SNE, and applied no dimension reduction technique, to the validation set. Both of these results produced less distinct clusters compared to t-SNE, with higher numbers of noise points (see below).

We used a Grid Search on a wide range of epsilons (x-axis), which represent the threshold distance between two neighboring points to classify points as within the same cluster, to extract OPTICS clusters (**see Figures below**). We compared the silhouette width (y-axis), which reflects how similar an observation is to those in its own cluster as compared to observations in other clusters, for each epsilon to find the optimal clustering. The numbers next to the points indicate the number of clusters found using that epsilon. Percent of noise points are showed by the colors of the points. Clusterings with less than two clusters were omitted in the figures. When using Principal Component Analysis to reduce dimensions (left), we found at most four clusters. When using no dimension reduction (right), we found at most three clusters. All of them had more than 50% of noise points. **Given the limited number of clusters with high proportion of noise points (individuals unable to be clustered, we felt that these two methods were less robust than OPTICS with t-SNE for dimension reduction.**

OPTICS on original features (no dimension reduction) produces limited number of features with high noise points

OPTICS using principal component analysis for dimension reduction produces limited number of clusters with high noise points


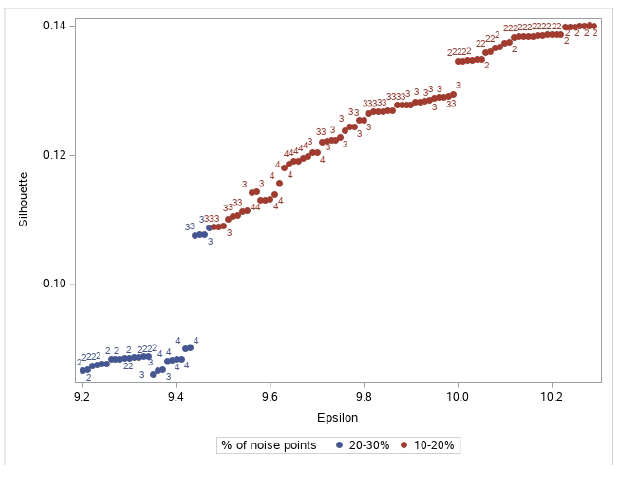

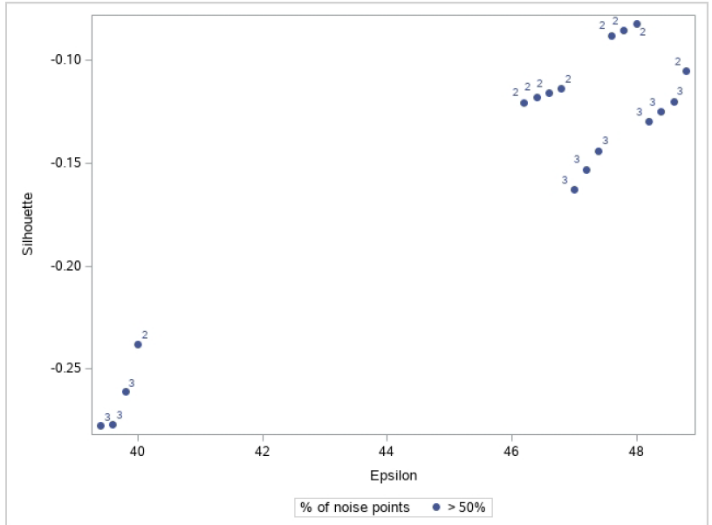


Epsilon = the threshold distance between two neighboring points to classify points as within the same cluster

Silhouette = How similar an observation is to those in its own cluster as compared to observations in other clusters

The numbers next to the points indicate the number of clusters found using that epsilon.

Percent of noise points are indicated by the colors of the points.

## Cluster Analysis

We used the Ordering Points To Identify the Clustering Structure (OPTICS) algorithm – a density-based clustering algorithm - on each t-SNE dimension-reduced data set. OPTICS was chosen based on its performance in prior work clustering a high-risk patient population. OPTICS requires a specified minimum number of nearest neighbors within a specified radius (ε) around each observation. Points located in relatively low-density regions have few “neighbors” and may be labeled as outliers. We set the minimum number of patients to be 0.5% in each 10k subset, or 50 veterans, to help ensure that clusters would not be so small as to be operationally insignificant. We also *a priori* restricted the number of noise points to be less than 50% to be operationally meaningful. We used the 10 training sets to find the optimal ε for the validation and test sets. To do so, we first specified a grid of ε values varying from 1 to 10 in increments of 0.1. We then calculated the average silhouette width for each ε value separately using each training set. We used Euclidean distance in the calculation of silhouette. Average silhouette width reflects how similar an observation is to those in its own cluster as compared to observations in other clusters. Next, we averaged the average silhouette values for each ε across training sets and chose the optimal ε as the value that maximized the average silhouette width. Lastly, we applied the optimal ε to cluster the validation set and test set using OPTICS.

## Identifying Subgroups

As described in the main text, we used a set of ridge regression models to estimate the contribution of all variables to each identified cluster, in order to label it. An example below provides an explanation:

The table below shows the standardized ridge regression coefficients of the 5 most important variables in the 5 psychobehavioral clusters. The first cluster featured by the highest amphetamine use, high drug abuse, outpatient substance use visits, cannabis use, and low sedative use compared to other clusters was labeled as a*mphetamine predominant*. Similarly, the second cluster having the most sedative use, more drug abuse, opioid use, inpatient psychiatric visits, and less amphetamine use was identified as *sedative predominant*. The third cluster presenting elevated opioid use, drug abuse, outpatient substance use visits, number of opioid abuse therapies, and younger age was labeled as *Opioid predominant*. The fourth cluster exhibiting high drug abuse, cannabis use, cocaine use, alcohol abuse, but low HIV/AIDS diagnosis was labeled as *polysubstance use - not otherwise specified*. On the contrary, the last cluster showing high psychoses diagnosis, number of antipsychotic medication, outpatient and inpatient psychiatric visits, but low opioid, sedative, cannabis, cocaine, amphetamine use was labeled as *psychoses without drug abuse*.

| **Variable** | **Polysubstance use - amphetamine predominant** | **Polysubstance use - sedative predominant** | **Polysubstance use - opioid predominant** | **Polysubstance use - not otherwise specified** | **Psychoses without drug abuse** | **Range** |
| --- | --- | --- | --- | --- | --- | --- |
| **Amphetamine Use** | 0.232 | -0.036 | -0.018 | -0.017 | -0.009 | 0.268 |
| **Sedative Use** | -0.039 | 0.211 | -0.015 | -0.016 | -0.012 | 0.251 |
| **Opioid Use** | 0.017 | 0.034 | 0.123 | -0.017 | -0.016 | 0.140 |
| **Psychoses** | 0.006 | 0.015 | -0.003 | 0.007 | 0.093 | 0.096 |
| **Cannabis Use** | 0.021 | 0.027 | 0.014 | 0.079 | -0.010 | 0.089 |
| **Drug Abuse** | 0.053 | 0.045 | 0.060 | 0.079 | -0.008 | 0.087 |
| **Cocaine Use** | 0.012 | 0.012 | -0.013 | 0.069 | -0.011 | 0.082 |
| **Outpatient substance use visits** | 0.035 | 0.015 | 0.049 | 0.015 | -0.010 | 0.060 |
| **# of antipsychotics** | 0.009 | 0.016 | 0.008 | 0.009 | 0.065 | 0.056 |
| **Outpatient psychiatric visits** | 0.015 | 0.015 | 0.007 | 0.015 | 0.063 | 0.055 |
| **Inpatient psychiatric visits** | 0.010 | 0.029 | -0.013 | 0.015 | 0.030 | 0.042 |
| **Alcohol Abuse** | 0.017 | 0.021 | 0.014 | 0.035 | -0.004 | 0.038 |
| **# of opioid abuse therapies** | 0.008 | 0.016 | 0.025 | -0.009 | 0.004 | 0.033 |
| **Height median** | 0.002 | 0.005 | 0.008 | 0.007 | 0.018 | 0.016 |
| **HIV/AIDS** | -0.002 | -0.002 | -0.009 | -0.018 | -0.007 | 0.016 |
| **Age as of 1/1/2014** | -0.017 | -0.008 | -0.023 | -0.016 | -0.012 | 0.015 |
